# Effects of a Bioprocessed Soybean Meal Ingredient on the Intestinal Microbiota of Hybrid Striped Bass, *Morone chrysops x M. saxatilis*

**DOI:** 10.3390/microorganisms9051032

**Published:** 2021-05-11

**Authors:** Emily Celeste Fowler, Prakash Poudel, Brandon White, Benoit St-Pierre, Michael Brown

**Affiliations:** 1 Department of Animal Science, South Dakota State University, Brookings, SD 57007, USA; Emily.Fowler@sdstate.edu (E.C.F.); Prakash@Himalayandogchew.com (P.P.); 2 Department of Natural Resource Management, South Dakota State University, Brookings, SD 57007, USA; brandon@prairieaquatech.com

**Keywords:** hybrid striped bass, microbiome, bacteria, bioprocessed soybean meal

## Abstract

The hybrid striped bass (*Morone chrysops x M. saxatilis*) is a carnivorous species and a major product of US aquaculture. To reduce costs and improve resource sustainability, traditional ingredients used in fish diets are becoming more broadly replaced by plant-based products; however, plant meals can be problematic for carnivorous fish. Bioprocessing has improved nutritional quality and allowed higher inclusions in fish diets, but these could potentially affect other systems such as the gut microbiome. In this context, the effects of bioprocessed soybean meal on the intestinal bacterial composition in hybrid striped bass were investigated. Using high-throughput sequencing of amplicons targeting the V1–V3 region of the 16S rRNA gene, no significant difference in bacterial composition was observed between fish fed a control diet, and fish fed a diet with the base bioprocessed soybean meal. The prominent Operational Taxonomic Unit (OTU) in these samples was predicted to be a novel species affiliated to *Peptostreptococcaceae*. In contrast, the intestinal bacterial communities of fish fed bioprocessed soybean meal that had been further modified after fermentation exhibited lower alpha diversity (*p* < 0.05), as well as distinct and more varied composition patterns, with OTUs predicted to be strains of *Lactococcus lactis*, *Plesiomonas shigelloides*, or *Ralstonia pickettii* being the most dominant. Together, these results suggest that compounds in bioprocessed soybean meal can affect intestinal bacterial communities in hybrid striped bass.

## 1. Introduction

As a result of the growing market demand for seafood and the depletion of wild fish populations, the aquaculture industry has considerably expanded over the last few decades [1]. Of the various fish species available for production, the hybrid striped bass has proven to be well suited for aquaculture because of its high growth performance, survival, and disease resistance, as well as its ability to be reared under a number of different culture systems and conditions [2]. The hybrid striped bass is the result of crossing female white bass (*Morone chrysops*) with male striped bass (*M. saxatilis*), and its higher performance compared to its parent species is attributed to hybrid vigor [2]. The success of the hybrid striped bass has been well illustrated by the rapid expansion of its global production, starting at five metric tons in 1986, increasing by 36.8-fold to 184 metric tons in 1987, then peaking at 6203 metric tons by 2005 [3]. World production levels then fluctuated between 3764 metric tons and 5884 metric tons between 2006 and 2016 [3]. Hybrid striped bass has become one of the leading aquaculture industries in the United States, behind channel catfish (*Ictalurus punctatus*), Atlantic salmon (*Salmo salar*), and rainbow trout (*Oncorhynchus mykiss*) [4].

As with other intensive animal production systems, minimizing operating costs represents one of the main challenges faced by aquaculture producers, with purchasing of dietary ingredients generally representing a major expense. In the case of hybrid striped bass production, for instance, nutrition costs have been estimated to make up approximately 40% of total variable costs [5]. As fishmeal remains an important source of dietary protein in aquaculture diets, its increasing market price and decreasing availability have been particularly problematic [6,7]. In response to this challenge, fishmeal is being replaced by more economically and environmentally sustainable sources of dietary protein in the formulation of aquaculture diets; inclusion of fishmeal in salmonid diets, for example, has decreased from 50% in the 1990s to 15% by 2012 [8].

Of the available alternatives to fishmeal, plant-based protein ingredients such as soybean meal have become an attractive substitute because of their availability and lower cost [9]. However, the inclusion of soybean meal in carnivorous fish diets needs to be limited because of the presence of anti-nutritional factors, as these reduce digestibility and increase digestive tract inflammation, which is associated with intestinal enteritis [10]. Another concern of higher inclusion of plant-based protein sources in carnivorous fish diets is their higher carbohydrate content; carbohydrate levels need to be low enough to avoid negative effects on digestion and gut physiology. One effective solution to these problems has been the production of soy protein concentrate, a feed ingredient generated by the extraction of carbohydrates from soybean byproducts using ethanol [11]. Another approach involves the use of bioprocessing, a biotechnological strategy that aims to generate value-added products by treatment of a substrate with biocatalysts such as enzymes, microorganisms (bacteria and yeast or other fungi), or cells cultured from plants or animals. In the case of feed ingredients, microbial utilization of a substrate can effectively neutralize undesirable compounds, such as non-starch polysaccharides, protease inhibitors, lectins, saponins, phytic acid, phytoestrogens, and allergens. While bioprocessing has permitted higher inclusion of soybean meal in fish and livestock diets [12], efforts are still ongoing to increase the effectiveness of the procedure, optimize production scale-up, as well as improve the quality of the final product and/or custom tailor its composition to better suit specific areas of animal production.

For carnivorous fish, such as the hybrid striped bass, one unintentional consequence of including plant-based protein ingredients in diets may be changing the composition of the gut microbiome. Gut microbial communities have been shown to be important for the health and nutrition of a wide variety of host species, including fish [13,14]. Indeed, they promote the development and regulation of immune defenses, compete against pathogenic bacteria, and produce short-chain fatty acids from substrates that host enzymes are unable to digest [15,16,17]. In fish, the gut environment can be colonized as early as the larval stage, a process that can be modulated by factors such as diet, season, stage of development, and habitat [18]. Generally, the most abundant phyla in the fish gastrointestinal tract tend to be Proteobacteria followed by Firmicutes and Bacteroidetes, but this can vary depending on the trophic level of the host [19,20].

Considering the importance of the hybrid striped bass in the aquaculture industry, the composition of its gut microbiome in healthy individuals has remained mostly unexplored. Indeed, in contrast to salmon [21,22,23,24,25], trout [26,27,28,29,30,31,32], and catfish [33,34,35,36,37,38], no published studies are currently available on hybrid striped bass or its parental strains using DNA sequencing-based methods; culture-independent approaches using high throughput Next-Generation sequencing platforms have been established as the gold standard for the analysis of microbiomes. Using a culturing approach, *Aeromonas hydrophila* was identified as a dominant species in the gut of striped bass [39,40,41,42] and hybrid striped bass [43]. Considering the limited scope of culture-dependent techniques for analysis of gut microbial environments and that *A. hydrophila* has been recognized as a pathogen for a number of freshwater fish species [44], it can be concluded that the gut bacterial communities of the hybrid striped bass and of its parental fish species have yet to be investigated.

In this context, the study described in the present report aimed to determine and compare the intestinal bacterial community composition of hybrid striped bass fed diets that included bioprocessed soybean meal or modified bioprocessed soybean meal. These products were selected as test ingredients because of their lower cost compared to soy protein concentrate, as well as their potential to provide additional biotic properties. Together, results show that the composition of intestinal bacterial communities of hybrid striped bass fed bioprocessed soybean meal did not differ from the composition of a control diet that did not include the bioprocessed soybean meal. However, three diets that each included a different product variant of the bioprocessed soybean meal resulted in bacterial compositions that were very different.

## 2. Materials and Methods

### 2.1. Diet Formulations

The Control (CON) diet used in this study did not include soybean meal ingredients. It was designed from a documented formulation from the Agricultural Research Service digestibility database [45]. The treatment diets, which contained bioprocessed soybean meal or a subsequent product variant that was further modified after initial fermentation, were designed to replace 54.4% of wheat middling, 47.8% of the poultry meal, and 66.7% of the feather meal in a control diet (CON) (Table 1). Production of bioprocessed soybean meal consists of growing *Aureobasidium pullulans* on a pasteurized slurry of soybean meal in water for 4 to 5 days. The fungus converts sugars and oligosaccharides into fungal cell mass while also neutralizing anti-nutritional factors and other undesirable compounds. After completion of the fungal treatment, solids are recovered by centrifugation and then dried. The bioprocessed soybean meal ingredients resulting from post-fermentation treatments that were used in this study included three different fractions of the product (BP-F1, BP-F2, and BP-F3), bioprocessed soybean meal after an enzymatic treatment (BP-E), as well as bioprocessed soybean meal after an additional rinse or wash step (BP-W). The diets used in this study were formulated to be isocaloric and isonitrogenous, but some differences were detected by the proximate composition of nutrients (Table 2). While crude fat concentrations were similar among diets (8.13–8.99%), the CON diet had the lowest crude protein concentration (45.49%), compared to a range of 47.02–47.92% for the other diets, and it concomitantly had a higher nitrogen-free extract content (32.69% vs. 30.64–31.80%).

Dry ingredients were ground using a Fitzpatrick Commutator (Elmhurst, IL, USA) equipped with a 1.27 mm screen prior to blending. Milled ingredients were transferred to a ribbon mixer (Patterson Equipment, Toronto, ON, Canada), then blended for five minutes. The resulting homogenous feedstuff was extruded with an Extru-Tech E325 single-screw extruder (Sabetha, KS, USA), which was equipped with 2.5 mm die inserts to produce 3.2 mm diameter floating pellets. Extruded pellets were then dried with a conveyor oven drier (Colorado Mill Equipment, Canon City, CO, USA), screen sifted using a Rotex screener (Rotex Inc., Cincinnati, OH, USA), then lipid-coated with a Phlauer vacuum coater (A & J Mixing, Oakville, ON, Canada). Finally, diets were bagged for storage at room temperature until use. Feeds were manufactured at Prairie AquaTech (Brookings, SD, USA).

### 2.2. Feeding Trial

The feeding trial was run at the fish-holding laboratory in the Northern Plains Biostress Facility at South Dakota State University. Naïve, juvenile hybrid striped bass (*n* = 560; 17.83 ± 0.11 g; Keo Fish Farm, Keo, AR, USA) were randomly distributed at a density of 20 fish per tank, with 4 replicate tanks randomly assigned to each dietary treatment. All fish were fed the same ration of a fishmeal-based holding diet prior to the start of the trial, and then each tank was switched to its assigned experimental diets upon the start of the trial. Feed was hand fed and offered to satiety to each tank three times per day (08:00, 12:00, and 16:00) for 105 consecutive days, and the amount of feed consumed was recorded. The trial was conducted with a 4682 L recirculating aquaculture system (RAS), consisting of 28 tanks, each with a 114 L capacity. Tanks were each equipped with a “recirculating” drain which withdrew water from the subsurface, and a “sludge” drain which was affixed to the lowest point in the bottom at the center of the tank. Each tank also contained forced air diffusers fed by a blower, as well as half covers to minimize disturbance. The RAS was also equipped with a pump, bead filter, bag filter, UV filter, biofilter, solids settling sump, clarifying sump, water inlet float valve, and heater/chiller unit. Water temperature was maintained between 25 °C and 27 °C, dissolved oxygen was held at levels greater than 5 mg/L, and the range in pH was 7–8. Temperature, dissolved oxygen, and pH were monitored daily, while ammonia (NH_3_) and nitrite (NO_2_^−^) were monitored on average three times per week.

At the end of the study, randomly selected individual fish were euthanized using lethal levels of buffered MS-222, according to a protocol approved by the Institutional Animal Care and Use Committee (IACUC) (Approval Number 16-089A). Liver and viscera were collected by dissection from three randomly selected fish from each tank to calculate the hepatosomatic index (HSI = (liver weight/body weight) × 100) and viscerosomatic index (VSI = (viscera weight/body weight) × 100). Each fish used for microbiota analysis was randomly selected from an individual tank. Distal intestines were cut from the vent; then feces were recovered into a sterile tube by gently running forceps along the outside of the intestine. Collected feces were flash-frozen in liquid nitrogen, then stored at −80 °C until they were processed for bacterial community composition.

### 2.3. Microbial DNA Isolation and PCR Amplification

Microbial genomic DNA was isolated from intestinal samples by a repeated bead beating plus column method [46], which included the use of the QIAamp DNA Mini Kit (Qiagen, Hilden, Germany). One dissected intestine was used as starting material for each microbial genomic DNA preparation. Bead beating was performed twice for each DNA preparation, for a duration of 3 min at 3500 rpm for each repetition. The V1–V3 region of the bacterial 16S rRNA gene was targeted using the 27F forward [47] and 519R reverse [48] primer pair by PCR with the Phusion Taq DNA polymerase (Thermo Scientific, Waltham, MA, USA) under the following conditions: hot start (4 min, 98 °C), followed by 35 cycles of denaturation (10 s, 98 °C), annealing (30 s, 50 °C) and extension (30 s, 72 °C), then ending with a final extension period (10 min, 72 °C). A total of 5–30 ng of purified microbial genomic DNA was used per PCR reaction in a total reaction volume of 50 μL. PCR products were separated by agarose gel electrophoresis, and amplicons of the expected size (~500bp) were excised for gel purification using the QiaexII Gel extraction kit (Qiagen, Hilden, Germany). A negative control reaction (no DNA) was included for each series of PCR reactions; amplicon DNA from experimental samples was not recovered from sets of PCR reactions whose negative control showed detectable amplified DNA. For each sample, approximately 400 ng of amplified DNA were submitted to Molecular Research DNA (MRDNA, Shallowater, TX, USA), which performed all subsequent steps for Next-Generation sequencing, including indexing and library preparation, to generate overlapping paired-end reads with the Illumina MiSeq (2 × 300) platform.

### 2.4. Computational Analysis of PCR Generated 16S rRNA Amplicon Sequences

Unless specified, sequence data analysis was performed using custom-written Perl scripts. Raw bacterial 16S rRNA gene V1–V3 amplicon sequences were provided by Molecular Research DNA (MRDNA, Shallowater, TX, USA) as assembled contigs from overlapping MiSeq (2 × 300) paired-end reads from the same flow cell clusters. Reads were then selected to meet the following criteria: the presence of both intact 27F (forward) and 519R (reverse) primer nucleotide sequences, a length between 400 and 580 nt, and a minimum quality threshold of no more than 1% of nucleotides with a Phred quality score lower than 15 [49,50].

Following quality screens, sequence reads were aligned, then clustered into Operational Taxonomic Units (OTUs) at a genetic distance cutoff of 5% sequence dissimilarity [49,50]. OTUs were screened for DNA sequence artifacts using the following methods. Chimeric sequences were first identified with the ‘chimera.uchime’ [51] and ‘chimera.slayer’ [52] commands from the MOTHUR (version 1.44.1) open-source software package [53]. Secondly, the integrity of the 5′ and 3′ ends of OTUs was evaluated using a database alignment search-based approach; when compared to their closest match of equal or longer sequence length from the NCBI ‘nt’ database, as determined by BLAST [54], OTUs with more than five nucleotides missing from the 5′ or 3′ end of their respective alignments were discarded as artifacts. Single read OTUs were subjected to an additional screen, where only sequences that had a perfect or near-perfect match to a sequence in the NCBI ‘nt’ database were kept for analysis, i.e., that the alignment had to span the entire sequence of the OTU, and a maximum of 1% of dissimilar nucleotides was tolerated.

After removal of sequence chimeras and artifacts, OTUs were subjected to taxonomic assignments as follows: two general taxonomic level assignments (Phylum and Family) for all OTUs using RDP Classifier [55], and closest relative identification for select OTUs using BLAST queries [54]. Alpha diversity indices (Observed OTUs, Chao, Ace, Shannon, and Simpson) were determined using the ‘summary.single’ command from MOTHUR (version 1.44.1) [53] on a dataset subsampled to 5000 reads for each sample. Principle Coordinate Analysis (PCoA) for beta diversity was performed using the same rarefied dataset, by determining Bray–Curtis distances with the ‘summary.shared’ command followed by the ‘pcoa’ command in MOTHUR (version 1.44.1) [53].

### 2.5. Statistical Analysis

Normal distribution of fish performance data was first confirmed using the Shapiro-Wilk test, then an Analysis of Variance (ANOVA), with a Tukey’s HSD post hoc test for multiple comparisons, was performed for statistical analysis using JMP (Version 12, SAS Institute Inc., Cary, NC, USA). Comparisons of abundance for bacterial taxonomic groups and OTUs amongst different dietary treatments were performed in R (Version R-3.6.2) using the non-parametric test Kruskal–Wallis (command ‘kruskal.test’), followed by the Wilcoxon test (command ‘pairwise.wilcox.test’) for multiple pairwise comparisons, which included the Benjamini-Hochberg correction to control for false discovery rate. For alpha diversity indices, normal distribution of data was first confirmed using the Shapiro Wilk test (command ‘shapiro.test’), then comparison across the different diet groups was performed using ANOVA followed by Tukey’s range test for multiple comparisons; these tests were conducted using R (Version R-3.6.2). Statistical significance was set at *p* ≤ 0.05.

PERMANOVA (permutational multivariate analysis) was performed in R (Version R-3.6.2) using the command ‘adonis’, followed by the command ‘ pairwise.adonis’ to identify pairs of sample groups that were different. For all analyses, tests resulting in *p* ≤ 0.05 were considered significant. Analysis by LDA Effect Size (LEfSe) [56] was performed using a publicly available online implementation of the program (https://huttenhower.sph.harvard.edu/galaxy/ accessed on 16 October 2020).

### 2.6. Next-Generation Sequencing Data Accessibility

Raw sequence data are available from the NCBI Sequence Read Archive under Bioproject PRJNA718291.

## 3. Results

### 3.1. Feeding Trial Performance

Overall, all fish grew well across the seven dietary treatments, with 100% survival for the duration of the 105-day trial. While no difference in biomass gain per fish was detected among dietary treatments, the respective feed conversion ratios for all diets that included bioprocessed soybean meal were found to be improved since they were lower than for the CON diet (*p* < 0.05; Table 3).

### 3.2. Taxonomic Composition Analysis

**Figure 1:**
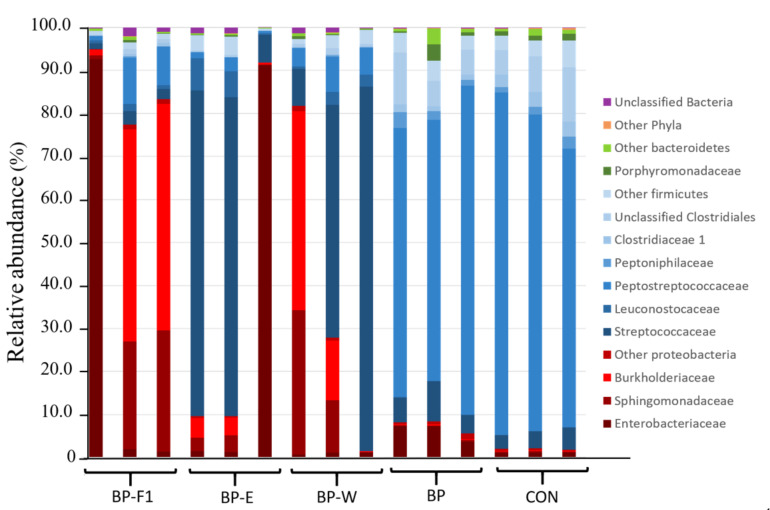
Taxonomic profiles at the phylum and family levels of intestinal bacterial communities of hybrid striped bass. Families belonging to the same phylum are represented by different shades of the same color: Firmicutes (blue), Bacteroidetes (green), and Proteobacteria (red).

A combined total of 15 samples from five of the dietary treatments were selected for investigating the intestinal bacterial composition of hybrid striped bass in response to the inclusion of bioprocessed soybean meal. In addition to samples with or without the inclusion of bioprocessed soybean meal (BP vs. CON), samples from three diets with modified bioprocessed soybean meal were also analyzed: BP-F1 and BP-W, which had the lowest FCR means, and BP-E, which had the highest digestibility of the seven diets (digestibility data not shown). A combined total of 302,427 high-quality sequence reads, ranging between 5561 and 87,780 sequence reads per sample (Supplementary Table S1), from the V1–V3 region of the 16S rRNA gene were generated from the five diets. Taxonomy-based composition analyses revealed that Firmicutes and Proteobacteria were the most abundant phyla across all samples (Table 4, Figure 1), with the former showing the highest representation across all diets except diet BP-F1. The respective abundances of the five main families from the phylum Firmicutes were all found to vary across dietary treatments (*p* ≤ 0.05). *Peptostreptococcaceae*, *Peptoniphilaceae*, and *Clostridiaceae* were numerically more abundant in the BP and CON samples. In contrast, *Leuconostocaceae* showed their highest representation in the BP-F1, BP-E, and BP-W groups, while *Streptococcaceae* were at higher levels in the BP-E and BP-W groups. Of the three main families of Proteobacteria identified in this study, only *Enterobacteriaceae* were found to vary across dietary treatments (*p* ≤ 0.05), with the highest levels observed in the BP-F1 and BP-E groups.

### 3.3. Alpha and Beta Diversity

**Figure 2:**
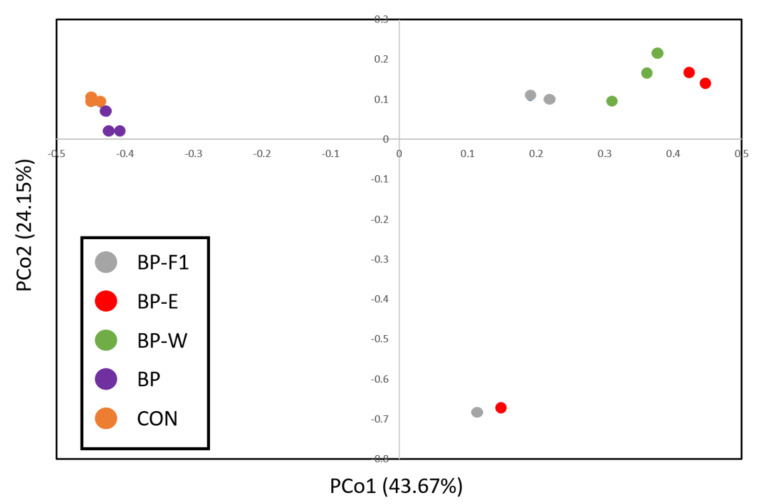
Comparison of intestinal bacterial communities in hybrid striped bass for five dietary treatments (BP-F1, BP-E, BP-W, BP, and CON). Principal Coordinate Analysis (PCoA) was performed based on the Bray–Curtis distance matrix. The x and y axes correspond to Principal Components 1 (PCo1) and 2 (PCo2), respectively, which together explained 67.82% of the variance. The PERMANOVA test supported the separation of samples into different groups (*p* = 0.001), but differences between groups could not be resolved by pairwise comparisons (*p* > 0.05).

Since taxonomic profiling indicated differences in composition associated with diets, OTU-level analyses were performed to gain further insight (Table 5). Based on the alpha diversity indices Observed OTUs, Ace, and Chao, dietary treatments appeared to fall into two distinct groups, with the group consisting of treatments BP-F1, BP-E, and BP-W having a lower number of OTUs compared to the group with BP and CON (*p* ≤ 0.05). Clustering of treatments into separate groups was consistent with PCoA (Figure 2) and supported by the PERMANOVA test (*p* = 0.001).

### 3.4. OTU Composition Analysis

**Figure 3:**
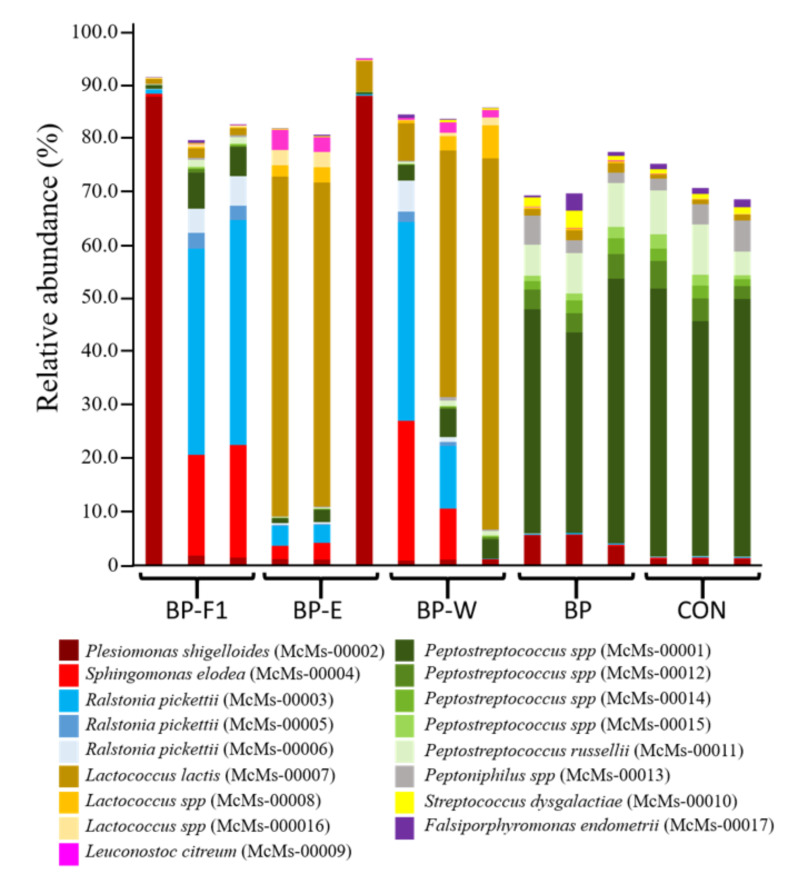
Histogram showing the relative abundance of the most highly represented intestinal OTUs in hybrid striped bass for five dietary treatments. OTUs showing 97% sequence identity or greater to their closest relative are represented by the full species name, while OTUs showing less than 97% identity to their closest relative are represented by their assigned genus.

Of the 1132 OTUs that were identified across all samples, the most abundant OTUs, defined as representing at least 1.0% of sequences in at least one set of samples, were further analyzed (Figure 3; Table 6). Eleven of these OTUs, one assigned to Proteobacteria and ten affiliated to Firmicutes, were found to vary across dietary treatments (*p* ≤ 0.05), and they exhibited composition patterns that were consistent with their respective taxonomic groups. For instance, SD_McMs-00002 was the most highly represented OTU of the family *Enterobacteriaceae*, representing 79.0–95.8% of sequence reads from this taxonomic group across all samples, and it accordingly was most abundant in diets BP-F1 and BP-E. Five of the OTUs of interest were assigned to *Peptostreptococcaceae* (SD_McMs-00001, SD_McMs-00011, SD_McMs-00012, SD_McMs-00014, and SD_McMs-00015), and their highest representation was in samples from diets BP and CON. SD_McMs-00001 was the most abundant OTU from this group, representing 2.86–4.42-times the combined read abundances from the other four *Peptostreptococcaceae* OTUs in each sample. Of the remaining Firmicutes OTUs that varied across dietary treatments, three were assigned to *Streptococcaceae* (SD_McMs-00007, SD_McMs-00010, and SD_McMs-00016). SD_McMs-00007 and SD_McMs-00016 were most closely related to *Lactococcus lactis*, and they were at their highest representation in diets BP-E and BP-W, while SD_McMs-00010 was most closely related to *Streptococcus dysgalactiae,* and it was most abundant in diets BP and CON. LEfse analysis was also used to identify biomarkers for the dietary treatments tested in the study; these consisted of 55 OTUs for CON, 38 OTUs for BPP, 17 OTUs for BP-E, four OTUs for BP-F1, and two OTUs for BP-W (Supplementary Figure S1). The eleven abundant OTUs identified as significantly different by Kruskal–Wallis (Table 6) were identified as biomarkers by LEfse.

## 4. Discussion

As a result of challenges such as cost and availability, the inclusion of fishmeal as a primary ingredient in aquaculture has become difficult to sustain [6]. Fishmeal use in aquaculture and livestock diets is also cause for ethical and social sustainability concerns, as increased demand for fishmeal would not only risk reducing the supply of fish available as food for humans but also promote overexploitation of species that are not for human consumption [57]. While other animal protein sources, such as poultry meal and feather meal, have served as suitable alternatives in aquaculture diets, there remains a critical need to find more economical replacement ingredients. While lower cost and availability make plant-based protein ingredients attractive alternatives, the presence of anti-nutritional factors such as non-starch polysaccharides, protease inhibitors, lectins, saponins, phytic acid, phytoestrogens, and allergens limits the extent to which they can be included in fish diets [58].

Thus, even if soybean meal provides a well-balanced amino acid profile, a favorable protein content, and lower amounts of anti-nutrients relative to other plant-based protein sources [59,60], its inclusion in diets of carnivorous species still needs to be restricted. Bioprocessing, i.e., modification of plant-based primary ingredients by microbial metabolism, has provided a solution to this problem. Indeed, fermentation of soybean meal into ‘bioprocessed’ soybean meal results in a product with an enhanced nutritional profile, as it is highly digestible and has a high protein content with increased lysine and methionine concentrations, while its anti-nutritional factor levels are greatly reduced [61].

One possible effect of the high inclusion of bioprocessed soybean meal in aquaculture diets is its potential impact on the composition of intestinal microbial communities. Considering their contributions to the health and nutrition of their host, alterations in the composition of symbiotic gut microbial communities could have unintended consequences on aquaculture production. In this context, the study presented in this report aimed at investigating the potential effects of bioprocessed soybean meal on the intestinal bacterial composition of aquaculture-raised hybrid striped bass. In addition to gaining further insight on an alternative feed ingredient with great potential for aquaculture, this report is also the first to provide insight on the gut microbiome of the hybrid striped bass using a culture-independent method.

The first observation was the absence of major differences in intestinal bacterial composition between fish fed a diet with the bioprocessed soybean meal (BP) and fish fed a diet without bioprocessed soybean meal (CON). Based on its limited sequence identity to its closest valid relative (*Peptostreptococcus* *russellii*, 91%), the main OTU (SD_McMs-00001) in these samples was predicted to correspond to a currently uncharacterized or uncultured bacterial species. Because of its taxonomic affiliation, SD_McMs-00001 would be predicted to utilize proteins as a main substrate. Indeed, strains of *P. russellii* were originally isolated from swine manure, and they were reported to produce elevated amounts of ammonia when grown in culture with various peptide-based ingredients [62]. This activity was interpreted as *P. russellii* playing an active role in the digestion and fermentation of proteinaceous material [63,64], which is consistent with the high protein content in carnivorous fish diets and the dietary treatments used in this study. Bacteria affiliated to the genus *Peptostreptococcus* have been identified as one of the main bacterial groups in rainbow trout [31] and proposed as an indicator taxon of fast-growing fish for this host [65]. This bacterial group was also reported to be well represented in aquaculture raised Arctic char (*Salvelinus alpinus*) [66]. Interestingly, many species of the genus *Peptostreptococcus* can increase the production of indoleacrylic acid and decrease the susceptibility of epithelial injury in mice [60]. Furthermore, research in humans has revealed that increased production of indoleacrylic acid could provide relief to inflammatory bowel disease [67]. Together, these previously published studies suggest that diets resulting in increased *P. russellii* may improve fish health by benefiting the host’s intestinal mucus layer.

Unexpectedly, the main intestinal OTUs in fish fed different post-fermentation modified versions of the bioprocessed soybean meal, i.e., diets BP-F1, BP-E, and BP-W, were very different from BP. The most abundant OTU in four of the six combined samples for BP-E and BP-W samples, SD_McMs-00007, was predicted to be a strain of *Lactococcus lactis* based on their high nucleotide sequence identity. *L. lactis* is known for its broad use in the food industry [68] because of its basic ability to utilize proteins and ferment carbohydrates into lactate. Strains of this species have been isolated from a number of distinct sources, including drain water and human vaginal samples [69,70], indicating that *L. lactis* is suited to many different types of environments. While it is not considered a typical resident of the gastrointestinal tract, *L. lactis* is capable of surviving in the gut environment [71,72], where it may interact with the mucus layer [73]. This species has been used as a probiotic in red sea bream (*Pagrus major*), resulting in a higher final weight, percent weight gain, and specific growth rate [74], as well as in olive flounder (*Paralichythys olivaceus*), where it was found to increase levels of growth-promoting metabolites, such as short-chain fatty acids, citrulline, taurine and vitamins [75].

The other two main OTUs in samples from diets BP-F1, BP-E, and BP-W may have represented potential pathogens. SD_McMs-00002 was 99% identical to *Plesiomonas* *shigelloides*, a bacterial species predominantly found in freshwater fish [40,76,77]. Notably, *P. shigelloides* was reported as a pathogen in cultured tilapia, with infected fish suffering tissue damage in the liver, spleen, kidney, heart, and intestine [78]. Since *P. shigelloides* has been reported as pathogenic in another report [76], future investigations will be required to gain further insight into the biological roles of SD_McMs-00002 and other related strains to determine whether this species represents a pathogen or a commensal in the hybrid striped bass. Similarly, SD_McMs-00003 was very closely related to *Ralstonia* *pickettii* (99%), also a bacterial species of the phylum Proteobacteria. While it has been proposed as a normal resident of the fish gastrointestinal tract [79], and it has been identified in soil and water samples [80], *R. pickettii* was also reported as a low virulence pathogen in certain cases of invasive infections in humans [81].

Based on the results presented in this report, the inclusion of bioprocessed soybean meal did not dramatically alter the intestinal bacterial composition of hybrid striped bass in the context of an aquaculture-based diet that consisted of a combination of fishmeal and alternative protein sources. However, inclusion of bioprocessed soybean meal that had been further processed after fermentation resulted in different and less consistent intestinal bacterial composition patterns. It will be of interest to further investigate these different effects of post-fermentation treated bioprocessed soybean meal on gut bacterial communities of aquaculture-raised fish in order to determine if the benefits of these alternative feed ingredients on performance are worth potential risks to gut microbiome function.

## Figures and Tables

**Table 1:** Experimental diet formulations used in the 105-day growth trial. All values are shown as g/(100 g dry matter).

Ingredient	Diet				
	BP-F1	BP-E	BP-W	BP	CON
BP-SBM Fraction #1 ^a^	25.00	0.00	0.00	0.00	0.00
BP-SBM Fraction #2 ^a^	0.00	0.00	0.00	0.00	0.00
BP-SBM Fraction #3 ^a^	0.00	0.00	0.00	0.00	0.00
BP-SBM + Enzyme ^a^	0.00	25.00	0.00	0.00	0.00
BP-SBM Base + Wash ^a^	0.00	0.00	25.00	0.00	0.00
BP-SBM Base ^a^	0.00	0.00	0.00	25.00	0.00
Blood Meal ^b^	2.50	2.50	2.50	2.50	2.50
Wheat Midds ^c^	10.00	10.00	10.00	10.00	21.92
Whole Cleaned Wheat ^d^	16.67	16.67	16.67	16.67	15.00
Poultry Meal ^e^	12.00	12.00	12.00	12.00	23.00
Feather Meal ^e^	2.50	2.50	2.50	2.50	7.50
Fish Meal ^f^	10.00	10.00	10.00	10.00	10.00
Vitamin Premix ^g^	1.25	1.25	1.25	1.25	1.25
Lysine ^h^	1.00	1.00	1.00	1.00	1.75
Methionine ^h^	0.50	0.50	0.50	0.50	0.50
Choline Chloride ^i^	0.58	0.58	0.58	0.58	0.58
Mineral Premix ^j^	0.75	0.75	0.75	0.75	0.75
Stay C ^k^	0.25	0.25	0.25	0.25	0.25
Fish Oil ^l^	6.50	6.50	6.50	6.50	4.50
Dicalcium phosphate ^m^	0.50	0.50	0.50	0.50	0.50
Defatted SBM ^n^	10.00	10.00	10.00	10.00	10.00
Totals	100.0	100.0	100.0	100.0	100.0

BP-SBM: bioprocessed soybean meal.^a^ South Dakota State University, Brookings, SD; ^b^ Mason City By-Products, Mason City, IA; ^c^ Consumer Supply Distributing, Sioux City, IA; ^d^ Ag First Farmer’s Cooperative, Brookings, South Dakota; ^e^ Tyson Foods, Springdale, AR; ^f^ Special Select, Omega Protein, Houston, TX; ^g^ ARS 702 premix, Nelson and Sons, Murray, UT; ^h^ Pure Bulk, Roseburg, OR; ^i^ BalChem Corporation, New Hampton, NJ; ^j^ ARS 640 trace mix, Nelson and Sons, Murray, UT; ^k^ DSM Nutritional Products, Parsippany, NJ; ^l^ Viginia Prime Gold, Omega Protein, Houson, TX; ^m^ Feed Products Inc., St. Louis, MO; ^n^ South Dakota Soybean Processors, Volga, South Dakota.

**Table 2:** Proximate composition of diets used in growth study. All values are shown as g/(100 g dry matter).

Diet	Ash	Fat	Fiber	Protein	NFE
BP-F1	7.71	8.79	4.65	47.32	31.53
BP-F2	7.89	8.53	3.86	47.92	31.80
BP-F3	7.63	8.97	4.47	47.25	31.68
BP-E	7.71	8.99	5.33	47.32	30.64
BP-W	7.77	8.49	5.62	47.02	31.10
BP	8.07	8.13	5.27	47.65	30.88
CON	8.88	8.42	4.52	45.49	32.69

NFE: Nitrogen Free Extract.

**Table 3:** Mean of performance indices for each dietary treatment over a 15 week trial.

Diet	Gain ^1^	Consumption ^2^	FCR ^3^	VSI ^4^	HIS ^5^
BP-F1	128.2 ^a^	185.0 ^ab^	1.44 ^c^	8.85 ^a^	1.49 ^bc^
BP-F2	119.9 ^a^	178.5 ^b^	1.49 ^b^	8.61 ^a^	1.44 ^bc^
BP-F3	110.8 ^a^	179.6 ^b^	1.62 ^b^	9.09 ^a^	1.40 ^c^
BP-E	105.6 ^a^	163.5 ^b^	1.55 ^b^	9.28 ^a^	1.56 ^abc^
BP-W	126.6 ^a^	183.8 ^ab^	1.45 ^bc^	8.57 ^a^	1.44 ^bc^
BP	119.7 ^a^	184.0 ^ab^	1.54 ^bc^	9.32 ^a^	1.64 ^ab^
CON	109.8 ^a^	211.2 ^a^	1.93 ^a^	8.60 ^a^	1.76 ^a^

Significant differences (*p* < 0.05) are indicated by different superscripts within a given column. ^1^: Average weight (g)/fish; ^2^: Average total consumption (g, dry)/fish; ^3^: Feed Conversion Ratio; ^4^: Viscerosomatic index (VSI); ^5^: Hepatosomatic index (HSI).

**Table 4:** Mean relative abundance (%) of main bacterial phyla and families identified in the intestine of hybrid striped bass.

Taxonomic Affiliation	CON	BP-F1	BP-E	BP-W	BP	*p* Value
Firmicutes	95.32	12.83	61.53	61.30	88.92	0.08
*Peptostreptococcaceae* ^#^	72.66 ^c^	6.90 ^abc^	1.67 ^a^	6.22 ^ab^	66.54 ^bc^	0.02
*Streptococcaceae* ^#^	4.09 ^ab^	2.29 ^a^	52.09 ^b^	49.18 ^b^	6.40 ^ab^	0.02
*Leuconostocaceae* ^#^	0.10 ^b^	1.05 ^a^	4.64^a^	2.10 ^a^	0.17 ^ab^	0.03
*Peptoniphilaceae* ^#^	1.90 ^b^	0.22 ^ab^	0.07^a^	0.23 ^ab^	2.35 ^b^	0.02
*Clostridiaceae* 1 ^#^	3.27 ^d^	0.44 ^abc^	0.06 ^b^	0.25 ^abc^	1.38 ^cd^	0.01
unclass. Clostridiales ^x^	8.89	0.78	0.29	0.84	7.94	-
Other Firmicutes ^x^	4.42	1.16	2.70	2.47	4.15	-
Proteobacteria	2.00	85.23	37.04	37.03	7.43	0.06
*Enterobacteriaceae* ^#^	1.12 ^c^	31.99 ^a^	31.28 ^ac^	1.02 ^bc^	6.10 ^a^	0.03
*Sphingomonadaceae*	0.30	18.04	2.46	15.29	0.25	0.26
*Burkholderiaceae*	0.24	34.43	3.01	20.06	0.26	0.11
Other Proteobacteria ^x^	0.35	0.77	0.29	0.65	0.83	-
Bacteriodetes	2.25	0.79	0.49	0.74	3.31	0.06
*Porphyromonadaceae*	1.25	0.28	0.18	0.29	1.63	0.06
Other Bacteroidetes ^x^	1.00	0.52	0.31	0.45	1.67	-
Other Bacteria ^x$^	0.42	1.14	0.94	0.93	0.34	-

Mean relative abundance of taxonomic groups is presented as a percentage (%) of the total number of analyzed reads per sample. Please see Supplementary Table S2 for standard errors of the means. ^#^ Taxa showing a statistically significant difference by the Kruskal–Wallis sum rank test (*p* < 0.05). Different superscripts in the same row indicate that groups are significantly different by the Wilcoxon test for multiple pairwise comparisons. ^x^ Statistical test not performed because of group heterogeneity. ^$^ Other bacteria include Actinobacteria, Spirochaetes, Fusobacteria, Acidobacteria, Planctomycetes, as well as unclassified bacteria.

**Table 5:** Observed OTUs and alpha-diversity indices in five dietary treatment groups. Values are shown as means.

Index	CON	BP-F1	BP-E	BP-W	BP	*p*-Value
Observed OTUs ^#^	266.67 ^b^	172.67 ^a^	134.00 ^a^	169.67 ^a^	301.67 ^b^	<0.001
Ace ^#^	799.75 ^b^	397.48 ^a^	305.62 ^a^	377.39 ^a^	725.13 ^b^	<0.001
Chao ^#^	550.36 ^b^	284.72 ^a^	269.85 ^a^	306.63 ^a^	545.64 ^b^	<0.001
Shannon	2.81	1.94	1.59	2.17	3.03	0.078
Simpson	0.24	0.40	0.53	0.32	0.20	0.287

^#^ Taxa showing a statistically significant difference by ANOVA (*p* < 0.05). Please see Supplementary Table S3 for standard errors of the means. Different superscripts in the same row indicate that groups are significantly different by the Tukey’s range test for multiple pairwise comparisons.

**Table 6:** Mean relative abundance of the main bacterial OTUs identified in hybrid striped bass. Abundance is presented as a percentage (%) of the total number of analyzed reads per sample.

OTUs	CON	BP-F1	BP-E	BP-W	BP	*p*-Value	Closest Taxon (id%)
Proteobacteria							
SD_McMs-00002 ^#^	1.03 ^ab^	30.28 ^a^	29.96 ^ab^	0.84 ^b^	4.82 ^a^	0.05	*Pl. shigelloides* (99%)
SD_McMs-00003	0.18	27.22	2.44	16.41	0.21	0.09	*R. pickettii* (99%)
SD_McMs-00004	0.23	13.54	1.90	11.97	0.18	0.29	*Sp. elodea* (99%)
SD_McMs-00005	0.02	1.92	0.14	0.92	0.01	0.16	*R. pickettii* (98%)
SD_McMs-00006	0.03	3.39	0.26	2.24	0.03	0.24	*R. pickettii* (99%)
Firmicutes							
SD_McMs-00001 ^#^	47.61 ^a^	4.29 ^abc^	1.15 ^c^	3.91 ^bc^	43.13 ^ab^	0.02	*Ps. russellii* (91%)
SD_McMs-00007 ^#^	0.86 ^a^	1.35 ^ab^	43.47 ^b^	41.01 ^b^	1.59 ^ab^	0.02	*La. lactis* (100%)
SD_McMs-00008	0.16	0.16	1.72	3.19	0.34	0.08	*La. lactis* (96%)
SD_McMs-00009 ^#^	0.03 ^a^	0.10 ^ab^	2.25 ^b^	1.20 ^b^	0.10 ^ab^	0.02	*Le. citreum* (100%)
SD_McMs-00010 ^#^	0.98 ^c^	0.05 ^a^	0.10 ^ac^	0.29 ^abc^	1.97 ^bc^	0.02	*St. dysgalactiae* (100%)
SD_McMs-00011 ^#^	7.41 ^a^	0.86 ^ab^	0.11 ^b^	0.75 ^ab^	7.20 ^a^	0.02	*Ps. russellii* (99%)
SD_McMs-00012 ^#^	3.99 ^a^	0.31 ^ab^	0.07 ^b^	0.27 ^ab^	3.98 ^a^	0.02	*Ps. russellii* (91%)
SD_McMs-00013 ^#^	3.97 ^a^	0.28 ^b^	0.11 ^b^	0.37 ^ab^	3.30 ^ab^	0.02	*Pe. stercorisuis* (89%)
SD_McMs-00014 ^#^	1.98 ^bc^	0.16 ^ac^	0.04 ^a^	0.17 ^ab^	2.32 ^b^	0.02	*Ps. russellii* (94%)
SD_McMs-00015 ^#^	1.81 ^a^	0.17 ^ab^	0.04 ^b^	0.10 ^ab^	1.51 ^a^	0.02	*Ps. russellii* (94%)
SD_McMs-00016 ^#^	0.02 ^c^	0.31 ^ab^	1.96 ^b^	0.72 ^ab^	0.04 ^ac^	0.03	*La. lactis* (96%)
Bacteroidetes							
SD_McMs-00017	1.18	0.25	0.16	0.27	1.42	0.06	*F. endometrii* (99%)

^#^ OTUs showing a statistically significant difference by the Kruskal-Wallis sum rank test (*p* < 0.05). Different superscripts in the same row indicate that groups are significantly different by the Wilcoxon test for multiple pairwise comparisons. Please see Supplementary Table S4 for standard errors of the means and Supplementary Table S1 for a complete list of OTUs and their respective abundances. Abbreviations: *F.*: *Falsiporphyromonas*; *La.*: *Lactococcus*; *Le.*: *Leuconostoc*; *Pe.*: *Peptoniphilus*; *Ps.*: *Peptostreptococcus*; *Pl.*: *Plesiomonas*; *R.*: *Ralstonia*; *Sp.*: *Sphingomonas*; *St.*: *Streptococcus*.

## Data Availability

Raw sequence data are available from the NCBI Sequence Read Archive under Bioproject PRJNA718291.

## Supplementary Materials

The following are available online at https://www.mdpi.com/article/10.3390/microorganisms9051032/s1.
