# Supplementary material for: Effects of a Bioprocessed Soybean Meal Ingredient on the Intestinal Microbiota of Hybrid Striped Bass, Morone chrysops x M. saxatilis
Source: Microorganisms. 2021 May 11;9(5):1032. doi: 10.3390/microorganisms9051032 (PMC8151853; doi:10.3390/microorganisms9051032)
Supplement: Supplementary file 1 [file microorganisms-09-01032-s001.zip › Fowler et al Supplementary Files_revised/Fowler et al HSB Supplementary Tables2-4_revised.docx]

**Supplementary Table 2.** Mean relative abundance (%) and standard error of the means of main bacterial taxonomic groups in hybrid striped bass.

| **Taxonomic Affiliation** | **BP-F1** | **BP-E** | **BP-W** | **BP** | **CON** |
| --- | --- | --- | --- | --- | --- |
| **Firmicutes** | 12.83 ± 2.22 | 61.53 ± 13.41 | 61.30 ± 12.12 | 88.92 ± 1.29 | 95.32 ± 0.19 |
| Peptostreptococcaceae^#^ | 6.90^abc^ ± 1.48 | 1.67^a^ ± 0.39 | 6.22^ab^ ± 0.56 | 66.54^bc^ ± 2.49 | 72.66^c^ ± 2.15 |
| Streptococcaceae^#^ | 2.29^a^ ± 0.25 | 52.09^b^ ± 11.41 | 49.18^b^ ± 11.05 | 6.40^ab^ ± 0.75 | 4.09^ab^ ± 0.28 |
| Leuconostocaceae^#^ | 1.05^a^ ± 0.15 | 4.64^a^ ± 1.09 | 2.10^a^ ± 0.41 | 0.17^ab^ ± 0.01 | 0.10^b^ ± <0.01 |
| Peptoniphilaceae^#^ | 0.22^ab^ ± 0.06 | 0.07^a^ ± 0.01 | 0.23^ab^ ± 0.03 | 2.35^b^ ± 0.35 | 1.90^b^ ± 0.21 |
| Clostridiaceae 1^#^ | 0.44^abc^ ± 0.09 | 0.06^b^ ± 0.01 | 0.25^abc^ ± 0.03 | 1.38^cd^ ± 0.09 | 3.27^d^ ± 0.10 |
| unclassified Clostridiales^&^ | 0.78 ± 0.14 | 0.29 ± 0.06 | 0.84 ± 0.11 | 7.94 ± 1.03 | 8.89 ± 1.01 |
| Other Firmicutes^&^ | 1.16 ± 0.10 | 2.70 ± 0.57 | 2.47 ± 0.34 | 4.15 ± 0.24 | 4.42 ± 0.46 |
| **Proteobacteria** | 85.23 ± 2.57 | 37.04 ± 13.70 | 37.03 ± 11.82 | 7.43 ± 0.44 | 2.00 ± 0.05 |
| Enterobacteriaceae^#^ | 31.99^a^ ± 15.16 | 31.28^ac^ ± 14.97 | 1.02^bc^ ± 0.07 | 6.10^a^ ± 0.60 | 1.12^c^ ± 0.01 |
| Sphingomonadaceae | 18.04 ± 4.30 | 2.46 ± 0.57 | 15.29 ± 4.88 | 0.25 ± 0.02 | 0.30 ± 0.01 |
| Burkholderiaceae | 34.43 ± 8.30 | 3.01 ± 0.66 | 20.06 ± 6.83 | 0.26 ± 0.01 | 0.24 ± 0.01 |
| Other Proteobacteria^&^ | 0.77 ± 0.17 | 0.29 ± 0.07 | 0.65 ± 0.18 | 0.83 ± 0.13 | 0.35 ± 0.04 |
| **Bacteriodetes** | 0.79 ± 0.15 | 0.49 ± 0.08 | 0.74 ± 0.15 | 3.31 ± 1.03 | 2.25 ± 0.18 |
| Porphyromonadaceae | 0.28 ± 0.09 | 0.18 ± 0.05 | 0.29 ± 0.08 | 1.63 ± 0.53 | 1.25 ± 0.09 |
| Other Bacteroidetes^&^ | 0.52 ± 0.06 | 0.31 ± 0.03 | 0.45 ± 0.07 | 1.67 ± 0.50 | 1.00 ± 0.16 |
| **Other Bacteria^&$^** | 1.14 ± 0.28 | 0.94 ± 0.22 | 0.93 ± 0.19 | 0.34 ± 0.01 | 0.42 ±0.08 |

**^#^** Taxa showing a statistically significant difference by the Kruskal-Wallis sum rank test (*P* < 0.05).

Different superscripts in the same row indicate that groups are significantly different by the Wilcoxon test for multiple pairwise comparison.

**^&^** Statistical test not performed because of group heterogeneity.

**$** Other bacteria include Actinobacteria, Cyanobacteria, Spirochaetes, Fusobacteria, Acidobacteria, Planctomycetes as well as unclassified bacteria.

**Supplementary Table 3.** Observed OTUs and α-diversity indices in five dietary treatment groups. Values represent means and standard error of the means.

| **Index** | **BP-F1** | **BP-E** | **BP-W** | **BP** | **CON** |
| --- | --- | --- | --- | --- | --- |
| Observed OTUs^#^ | 172.67^a^ ± 15.18 | 134.00^a^ ± 12.47 | 169.67^a^ ± 7.20 | 301.67^b^ ± 8.02 | 266.67^b^ ± 2.68 |
| Ace^#^ | 397.48^a^ ± 42.29 | 305.62^a^ ± 16.44 | 377.39^a^ ± 15.53 | 725.13^b^ ± 7.59 | 799.75^b^ ± 22.22 |
| Chao^#^ | 284.72^a^ ± 28.13 | 269.85^a^ ± 16.56 | 306.63^a^ ± 9.26 | 545.64^b^ ± 17.78 | 550.36^b^ ± 17.13 |
| Shannon | 1.94 ± 0.29 | 1.59 ± 0.23 | 2.17 ± 0.12 | 3.03 ± 0.08 | 2.81 ± 0.04 |
| Simpson | 0.40 ± 0.09 | 0.53 ± 0.06 | 0.32 ± 0.04 | 0.20 ± 0.01 | 0.24 ± 0.01 |

**^#^** Taxa showing a statistically significant difference by ANOVA (*P* < 0.05).

Different superscripts in the same row indicate that groups are significantly different by the Tukey’s range test for multiple pairwise comparison.

**Supplementary Table 4.** Mean relative abundance and standard error of the means of main intestinal bacterial OTUs in hybrid striped bass. Abundance is presented as a percentage (%) of the total number of analyzed reads per sample.

| **OTUs** | **BP-F1** | | **BP-E** | **BP-W** | **BP** | **CON** | **Closest valid taxon (id%)** |
| --- | --- | --- | --- | --- | --- | --- | --- |
| **Proteobacteria** |  |  | |  |  |  |  |
| SD_McMs-00002^#^ | 30.28^a^ ± 14.40 | 29.96^ab^ ± 14.49 | | 0.84^b^ ± 0.03 | 4.82^a^ ± 0.33 | 1.03^ab^ ± 0.01 | *Plesiomonas shigelloides (99%)* |
| SD_McMs-00003 | 27.22 ± 6.63 | 2.44 ± 0.54 | | 16.41 ±5.49 | 0.21 ± 0.02 | 0.18 ± 0.02 | *Ralstonia pickettii (99%)* |
| SD_McMs-00004 | 13.54 ± 3.26 | 1.90 ± 0.45 | | 11.97 ± 3.82 | 0.18 ± 0.01 | 0.23 ± 0.01 | *Sphingomonas elodea (99%)* |
| SD_McMs-00005 | 1.92 ± 0.48 | 0.14 ± 0.03 | | 0.92 ± 0.29 | 0.01 ± <0.01 | 0.02 ± 0.01 | *Ralstonia pickettii (98%)* |
| SD_McMs-00006 | 3.39 ± 0.84 | 0.26 ± 0.06 | | 2.24 ± 0.90 | 0.03 ± 0.01 | 0.03 ± <0.01 | *Ralstonia pickettii (99%)* |
| **Firmicutes** |  |  | |  |  |  |  |
| SD_McMs-00001^#^ | 4.29^abc^ ± 0.92 | 1.15^c^ ± 0.29 | | 3.91^bc^ ± 0.37 | 43.13^ab^ ± 1.76 | 47.61^a^ ± 0.92 | *Peptostreptococcus russellii (91*%) |
| SD_McMs-00007^#^ | 1.35 ^ab^± 0.13 | 43.47^b^± 9.45 | | 41.01^b^ ± 9.15 | 1.59^ab^ ± 0.10 | 0.86^a^ ± 0.05 | *Lactococcus lactis (100%)* |
| SD_McMs-00008 | 0.16 ± 0.02 | 1.72± 0.42 | | 3.19 ± 0.83 | 0.34 ± 0.01 | 0.16 ± 0.02 | *Lactococcus lactis (96%)* |
| SD_McMs-00009^#^ | 0.10^ab^ ± 0.02 | 2.25^b^ ± 0.53 | | 1.20^b^ ± 0.24 | 0.10^ab^ ± 0.01 | 0.03^a^ ± <0.01 | *Leuconostoc citreum (100%)* |
| SD_McMs-00010^#^ | 0.05^a^ ± 0.01 | 0.10^ac^ ± 0.02 | | 0.29^abc^ ± 0.06 | 1.97^bc^ ± 0.34 | 0.98^c^ ± 0.05 | *Streptococcus dysgalactiae (100%)* |
| SD_McMs-00011^#^ | 0.86^ab^ ± 0.19 | 0.11^b^ ± 0.02 | | 0.75^ab^± 0.11 | 7.20^a^ ± 0.35 | 7.41^a^ ± 0.76 | *Peptostreptococcus russellii (99%)* |
| SD_McMs-00012^#^ | 0.31^ab^ ± 0.08 | 0.07^b^ ± 0.02 | | 0.27^ab^ ± 0.03 | 3.98^a^ ± 0.17 | 3.99^a^ ± 0.41 | *Peptostreptococcus russellii (91%)* |
| SD_McMs-00013^#^ | 0.28^b^ ± 0.05 | 0.11^b^ ± 0.02 | | 0.37^ab^ ± 0.06 | 3.30^ab^ ± 0.55 | 3.97^a^ ± 0.54 | *Peptoniphilus stercorisuis (89%)* |
| SD_McMs-00014^#^ | 0.16^ac^ ± 0.03 | 0.04^a^ ± 0.01 | | 0.17^ab^ ± 0.03 | 2.32^b^ ± 0.21 | 1.98^bc^ ± 0.19 | *Peptostreptococcus russellii (94%)* |
| SD_McMs-00015^#^ | 0.17^ab^ ± 0.04 | 0.04^b^ ± 0.01 | | 0.10^ab^ ± 0.01 | 1.51^a^ ± 0.16 | 1.81^a^ ±0.29 | *Peptostreptococcus russellii (94%)* |
| SD_McMs-00016^#^ | 0.31^ab^± 0.05 | 1.96^b^ ± 0.47 | | 0.72^ab^ ± 0.19 | 0.04^ac^ ± <0.01 | 0.02^c^ ± <0.01 | *Lactococcus lactis (96%)* |
| **Bacteriodetes** |  |  | |  |  |  |  |
| SD_McMs-00017^&^ | 0.25 ± 0.08 | 0.16 ± 0.04 | | 0.27 ± 0.08 | 1.42 ± 0.45 | 1.18 ± 0.08 | *Falsiporphyromonas endometrii (99%)* |
|  |  |  | |  |  |  |  |

**^#^** OTUs showing a statistically significant difference by the Kruskal-Wallis sum rank test (*P* < 0.05).

Different superscripts in the same row indicate that groups are significantly different by the Wilcoxon test for multiple pairwise comparison.
