# Supplementary material for: Effects of a Bioprocessed Soybean Meal Ingredient on the Intestinal Microbiota of Hybrid Striped Bass, Morone chrysops x M. saxatilis
Source: Microorganisms. 2021 May 11;9(5):1032. doi: 10.3390/microorganisms9051032 (PMC8151853; doi:10.3390/microorganisms9051032)
Supplement: Supplementary file 1 [file microorganisms-09-01032-s001.zip › Fowler et al Supplementary Files_revised/Fowler et al Supp Figure 1 Lefse analysis.pptx]

## Slide 1
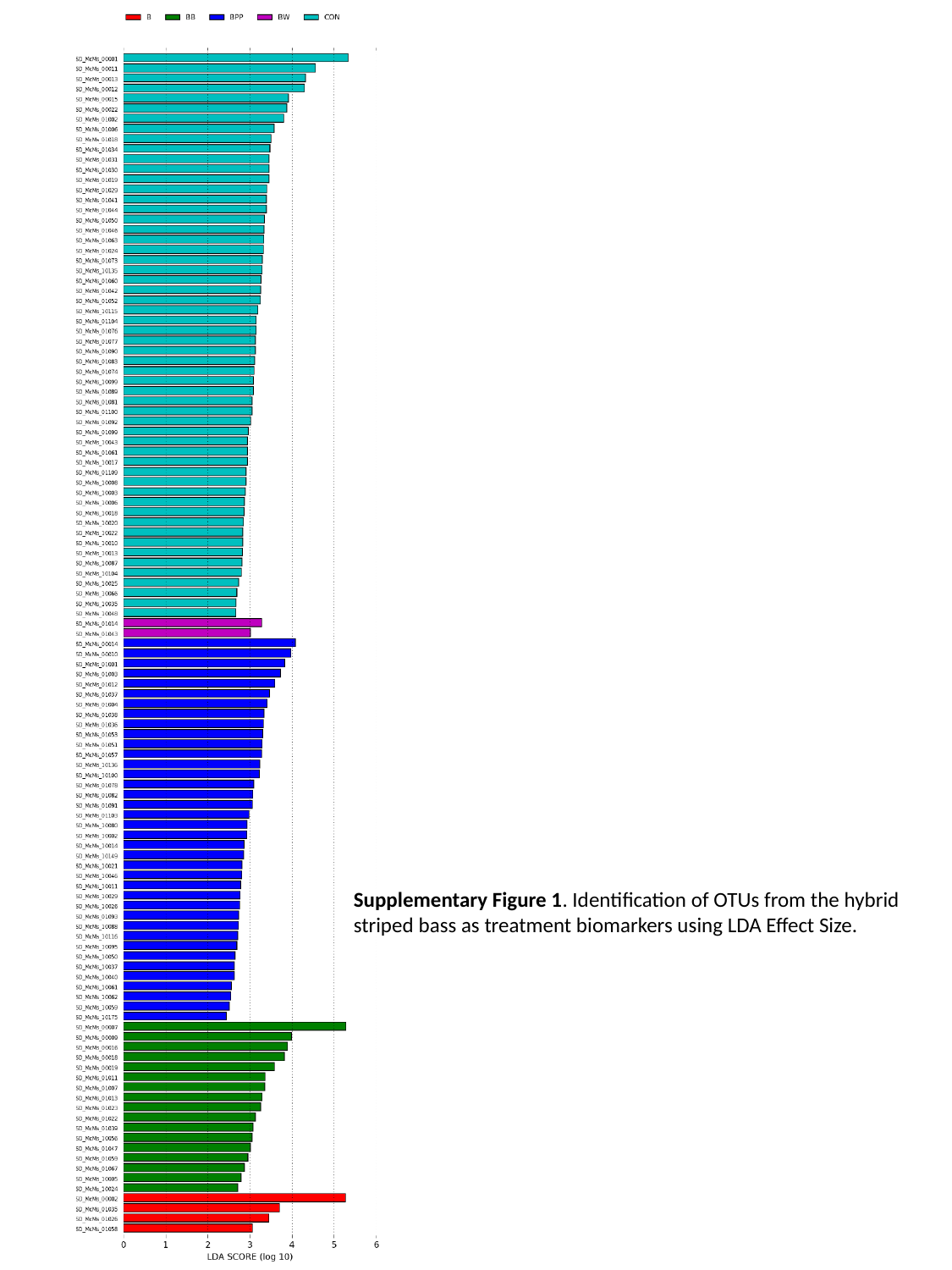

Supplementary Figure 1. Identification of OTUs from the hybrid striped bass as treatment biomarkers using LDA Effect Size.
